# Supplementary material for: The Predictive Value of the Cervical Consistency Index to Predict Spontaneous Preterm Birth in Asymptomatic Twin Pregnancies at the Second-Trimester Ultrasound Scan: A Prospective Cohort Study
Source: J Clin Med. 2020 Jun 8;9(6):1784. doi: 10.3390/jcm9061784 (PMC7356565; doi:10.3390/jcm9061784)
Supplement: Supplementary file 1 [file jcm-09-01784-s001.pdf]

Supplementary material

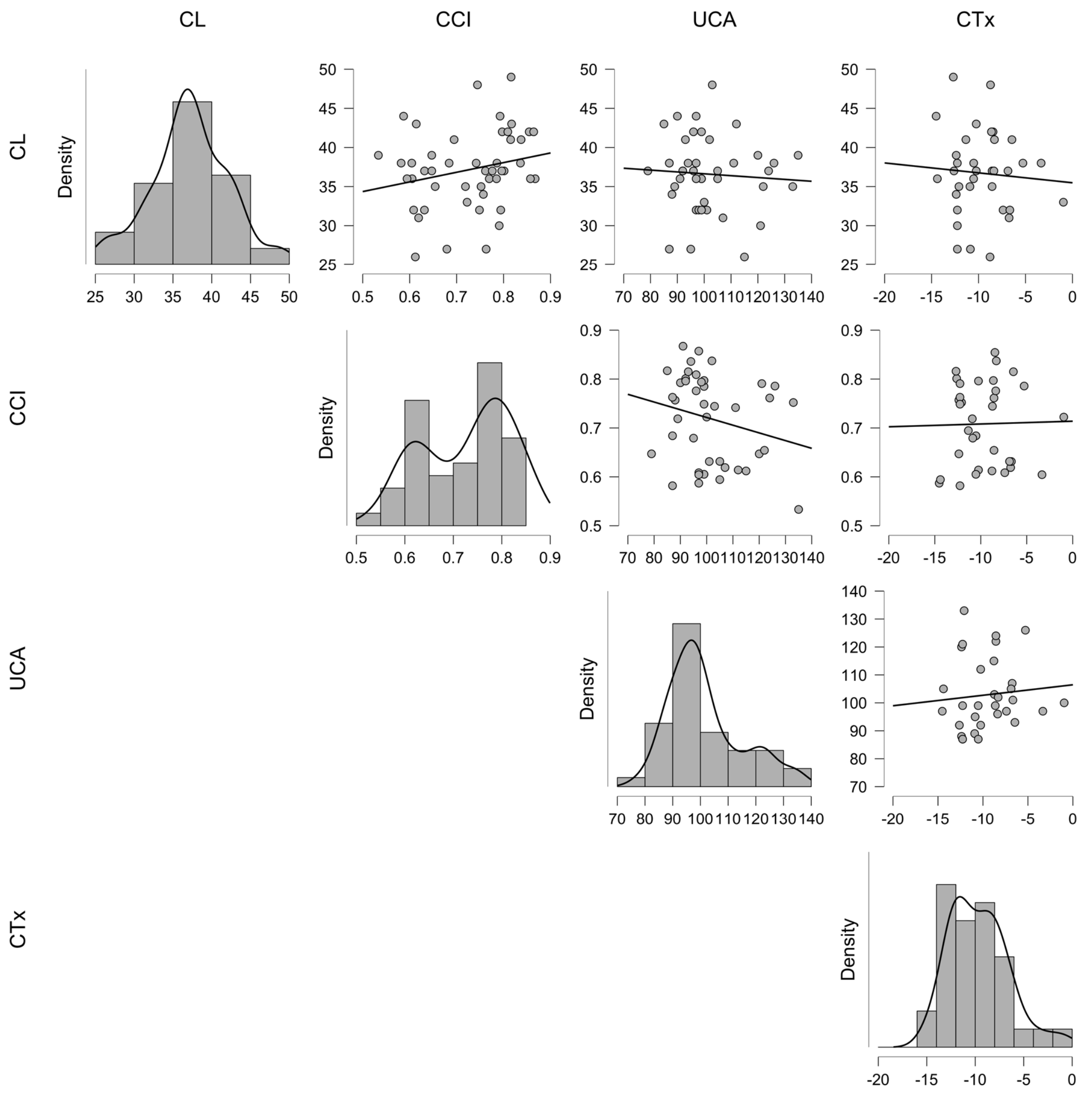

Figure S1. Distribution plots with density line indicated of the ultrasound derived markers at mid trimester with correlation plots between respective markers. Abbreviations: CL, cervical length; CCI, cervical consistency index; UCA, utero-cervical angle; CTx, cervical texture.

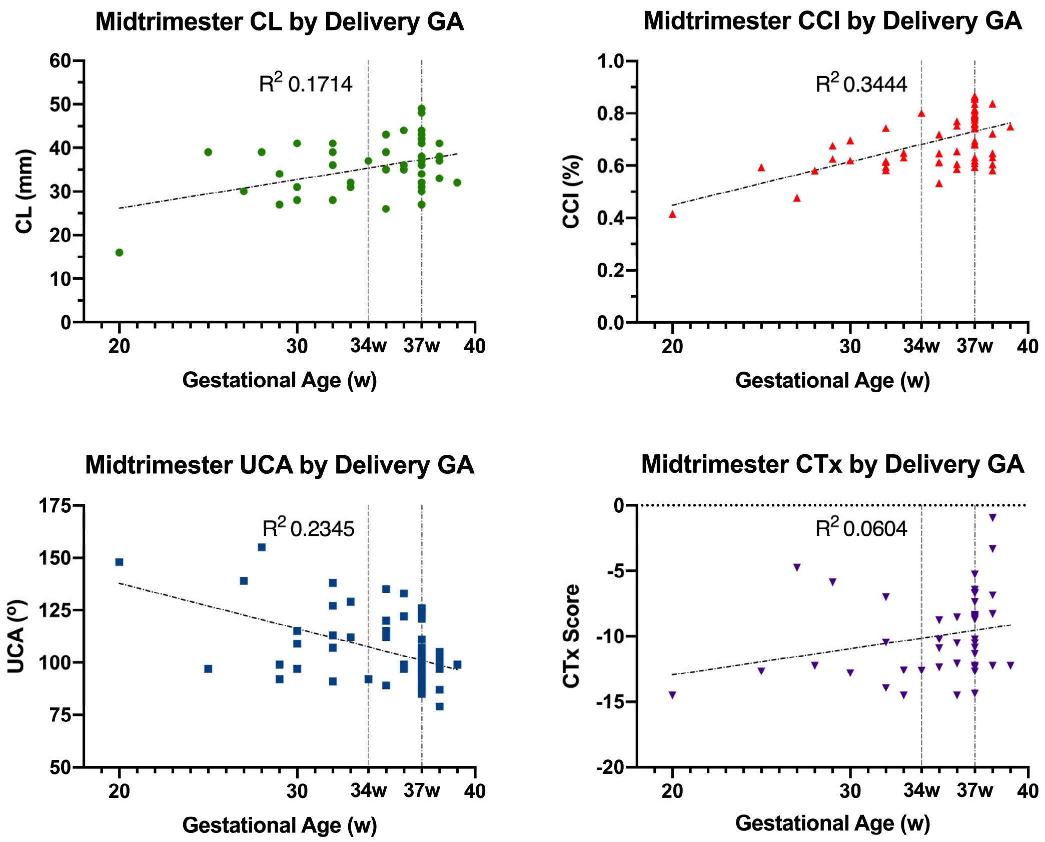

Figure S2. Plot of the ultrasound derived markers at mid trimester against the gestational age at delivery. Goodness-of-fit indicated by R^2 values. Abbreviations: CL, cervical length; CCI, cervical consistency index; UCA, utero-cervical angle; CTx, cervical texture.
